# Development and evaluation of a Loop Mediated Isothermal Amplification (LAMP) technique for the detection of hookworm (*Necator americanus*) infection in fecal samples

**DOI:** 10.1186/s13071-015-1183-9

**Published:** 2015-11-06

**Authors:** Robert Muriuki Mugambi, Eric L. Agola, Ibrahim N. Mwangi, Johnson Kinyua, Esther Andia Shiraho, Gerald M. Mkoji

**Affiliations:** Institute of Tropical Medicine and Infectious Diseases, Jomo Kenyatta University of Agriculture and Technology (JKUAT), P.O. Box 54840, 00200 Nairobi, Kenya; Center for Biotechnology Research and Development, Kenya Medical Research Institute (KEMRI), P.O Box 54840, 00200 Nairobi, Kenya; Department of Biochemistry, Jomo Kenyatta University of Agriculture and technology (JKUAT), P.O. Box 62000, 00200 Nairobi, Kenya

**Keywords:** Loop mediated isothermal amplification, ITS-2, *Necator americanus*, Kato-Katz

## Abstract

**Background:**

Hookworm infection is a major concern in sub-Saharan Africa, particularly in children and pregnant women. *Necator americanus* and *Ancylostoma duodenale* are responsible for this condition. Hookworm disease is one of the Neglected tropical diseases (NTDs) that are targeted for elimination through global mass chemotherapy. To support this there is a need for reliable diagnostic tools. The conventional diagnostic test, Kato-Katz that is based on microscopic detection of parasite ova in faecal samples, is not effective due to its low sensitivity that is brought about mainly by non-random distribution of eggs in stool and day to day variation in egg output. It is tedious, cumbersome to perform and requires experience for correct diagnosis. LAMP-based tests are simple, relatively cheap, offer greater sensitivity, specificity than existing tests, have high throughput capability, and are ideal for use at the point of care.

**Methods:**

We have developed a LAMP diagnostic test for detection of hookworm infection in faecal samples. LAMP relies on auto cycling strand displacement DNA synthesis performed at isothermal temperature by *Bst* polymerase and a set of 4 specific primers. The primers used in the LAMP assay were based on the second Internal Transcribed Spacer (ITS-2) region and designed using Primer Explorer version 4 Software. The ITS-2 region of the ribosomal gene (rDNA) was identified as a suitable target due to its low mutation rates and substantial differences between species. DNA was extracted directly from human faecal samples, followed by LAMP amplification at isothermal temperature of 63 °C for 1 h. Amplicons were visualized using gel electrophoresis and SYBR green dye. Both specificity and sensitivity of the assay were determined.

**Results:**

The LAMP based technique developed was able to detect *N. americanus* DNA in faecal samples. The assay showed 100 % specificity and no cross-reaction was observed with other helminth parasites (*S. mansoni, A. lumbricoides* or *T. trichiura)*. The developed LAMP assay was 97 % sensitive and DNA at concentrations as low as 0.4 fg were amplified.

**Conclusion:**

The LAMP assay developed is an appropriate diagnostic method for the detection of *N. americanus* DNA in human stool samples because of its simplicity, low cost, sensitivity, and specificity. It holds great promise as a useful diagnostic tool for use in disease control where infection intensities have been significantly reduced.

## Background

Hookworm infection in humans is caused by helminth nematode parasites *Necator americanus* and *Ancylostoma duodenale* and is transmitted through contact with contaminated soil. It is one of the most common chronic infections, with an estimated 740 million cases in the tropics and subtropics [1]. It is predominant among people living in poverty stricken areas [2]. The greatest number of hookworm cases occurs in Asia, followed by sub-Saharan Africa [1]. Globally, the most common hookworm species is *N. americanus*, while *A. duodenale* is more restricted geographically [3]. Hookworm infection is among the most important tropical diseases in humans. Disability-adjusted life years as a measure of the burden of disease reveals that this infection dominates African trypanosomiasis, dengue, Chagas’ disease, schistosomiasis, and leprosy [4]. The most damaging effects of hookworm infections include impaired physical, intellectual and cognitive development of children, increased mortality in pregnant women and their infants and reduced work capacity of adolescents and adults [5].

Hookworm has a direct lifecycle. The infective stage is a third stage larva that penetrates the skin upon contact with contaminated soil. While in the soil the third stage larvae are in a state of developmental arrest; development continues after it penetrates the skin of the human host [5]. The major clinical manifestation of hookworm infection is due to the consequences of chronic intestinal blood loss resulting in iron-deficiency leading to anaemia. Hypoalbuminemia develops when blood loss exceeds the intake and reserves of host iron and protein. The degree of iron-deficiency anaemia induced by hookworms depends on the species; *A. duodenale* causes greater blood loss than infection with *N. americanus* [4].

The formulation of effective control measures for hookworm is reliant on accurate diagnosis. Currently, the diagnosis of hookworms and other soil transmitted helminths is by the use of microscopy for the identification of ova in faeces and third-stage larvae (L3) through the copromiscroscopic technique, more specifically Kato-Katz. Kato-Katz method of diagnosis is widely accepted because it is technically simple and requires very minimal costs to perform [6]. Despite this, it is limited in terms of specificity because it cannot differentiate between ova of the two human hookworm species, specifically *N. americanus* and *A. duodenale*. It is also not sensitive enough to detect low infection in an individual. Other than its lack of sensitivity and specificity, microscopy based diagnostic technique is laborious, time consuming and requires a skilled individual for correct diagnosis.

Molecular based techniques that are dependent on Polymerase chain reaction (PCR) are available and are more sensitive and specific than the parasitological techniques. However, these methods are very expensive because of their requirement for costly equipment. Loop mediated isothermal amplification technique (LAMP) is a technique developed by Notomi et al. that amplifies DNA with high specificity, efficiency and rapidity under isothermal conditions. This nucleic acid amplification technique uses only one enzyme (*Bst* DNA polymerase) and is able to amplify large amounts of DNA within 30–60 min by auto-strand displacement DNA synthesis [7]. The technique has several advantages including; 1) the reaction takes place at isothermal temperatures between 60 °C and 65 °C, and therefore simple incubators, such as a water bath or block heater, are sufficient for the DNA amplification, 2) LAMP is more stable than PCR and real-time PCR, 3) Non-involvement of template DNA preparation and ability to generate 10^9^ copies of DNA are added benefits that make it more effective ,4) It is also easy to visualize the amplicons because in LAMP assay, as amplification of DNA continues there is the formation of pyrophosphates that causes turbidity and thus makes visualization easy [8]. The product can also be visualized directly using simple detection methods such as adding DNA binding dye, SYBR green. Performance of LAMP mainly relies on designing Primers that should be very specific. LAMP requires a minimum of 4 primers namely; F3 (Forward outer), B3 (Backward outer), FIP (Forward inner) and BIP (Backward inner) primers. Two extra loop primers, LF (Loop forward) and LB (Loop backward) can also be assimilated which hastens the reaction, hence taking a shorter time [9].

Loop mediated isothermal amplification has emerged as a powerful tool for point-of-care diagnostics and has been used in diagnosis of protozoan parasitic diseases such as *Plasmodium* [10], *S. haematobium* [11], African Trypanosomiasis [12], *S. japonicum* [13] and *Taenia* species [14] with high efficacy.

We have developed a loop mediated isothermal technique assay that is quick, sensitive, and specific with minimal equipment requirement for the diagnosis of hookworm (*N. americanus)* parasite in patient faecal samples.

## Methods

### Study approval and ethical considerations

This study was approved through the scientific and ethics committees of the Kenya Medical Research Institute (KEMRI). All the children found to be infected with hookworm or any of the soil-transmitted helminths received treatment with a single dose of 400 mg albendazole under the supervision of a clinician. Faecal sample collection for STH infection diagnosis is a routine procedure for parasitological investigations and does not pose any risks or cause any harm to participating children. The samples of the study participants were coded and any data that was obtained from them was stored in a password protected personal computer accessible only to the Principal Investigator.

### Faecal sample collection and parasitological examination

Each participating child provided a single faecal sample in a capped plastic polypot, and these were transported back to the field laboratory in an ice box, within 2 h of collection, where duplicate Kato-Katz slides were prepared on glass microscope slides according to the standard procedure and examined under a compound microscope at x400 magnification, within 1 h of sample preparation. All hookworm positive samples were used for DNA extraction.

### DNA extraction

Genomic DNA was extracted from freshly collected human stool samples using the QIAMP fast DNA stool mini kit from Qiagen, (catalog number 51604) following manufacturer instructions. Briefly, 0.2 g of stool sample was weighed and placed in 2 ml tubes. 1 mm of inhibit EX buffer was then added to the tube containing the faecal sample and vortexed for 1 min. The suspension was then heated for 5mins at 95 °C. In a new 1.5 ml tube, 15 μl proteinase K was added. The supernatant was then added into the tube containing 15 μl of proteinase K, and centrifuged. Two hundred microliters of buffer AL was then added and the mixture vortexed for 15 s. This was later incubated in a water bath for 10mins at 70 °C followed by addition of 200 μl of absolute ethanol and thorough mixing. From this mixture 600 μl of the lysate was pipetted into a spin column. This was centrifuged for 1 min at 14000 rpm and the filtrate discarded. At this point, 500 μl of buffer 1 (AW1) was added and centrifuged for 1 min and the filtrate discarded. 500 μl buffer 2(AW2) was then added and centrifuged for 3 mins at 14000 rpm. The filtrate was then discarded and 200 μl elution buffer (ATE) added and incubated at room temperature for 1 min and centrifuged for 1 min at 14000 rpm to elute the DNA. The DNA was stored at −20 °C until used.

### Primer design

LAMP Primers were designed using Primer Explorer version 4.0 software (http://primerexplorer.jp/elamp4.0.0). The internal transcribed spacer (ITS-2) gene was chosen for amplification because of the lower mutation rates and substantial differences between species. A total of 18 sequences of internal transcribed spacer 2 were selected from the National Centre for Biotechnology information (NCBI). The accession numbers of these sequences are KC896820.1-KC 896825.1, Y11734, AF217891, HQ452515, HQ455217, HQ452537-HQ452543 and AJ001599. These sequences were subjected to multiple sequence alignment (MSA) using a program called CLC main workbench version 7.0 and a consensus sequence was developed, from which five sets of primers were developed. The best set of primers was selected after being tested for their suitability and reliability. The four sets of primers (two outer and two inner primers) were designed to recognize six separate regions within the ITS-2 gene. Primers were validated using BLAST software (http://www.ncbi.nlm.nih.gov/BLAST) to ensure that they were specific to *N. americanus*. Primer sequences are listed in Table 1.

**Table 1:** Nucleotide sequences of the LAMP primers targeting the ITS-2 gene of *N. americanus*

Primer	Sequence
FIP	CAC TTA AAC GGG AAT TGC ACC GAA CGG TAC TTG CTC TG
BIP	GCA ACA TGTGCA CGC TGT TAA CAG TAT GCA CCG CTA TC
F3	AGT ATT GTT GAA CAC TGT TTTG T
B3	AAC AAC GAT ATG TTC ATG TCA T

Kato-Katz (gold standard)

### LAMP reaction

The LAMP reaction was performed as described by [7]. Briefly, in a total reaction volume of 18 μl, 40pmoles of each inner primer (FIP and BIP) and 5pmoles of each outer primer (F3 and B3), 12.5 μl of 2X LAMP buffer, 2.5 μl of Bst buffer, 1 unit of *Bst* polymerase and 2.0 μl of DNA template were added. Amplification was carried out at 63 °C for one hour, followed by reaction termination at 80 °C for 10 min.

### LAMP product detection

As part of the assay development, the LAMP products were initially detected by several methods, including electrophoresis on a 2.0 % agarose gel stained with 2 μl ethidium bromide and photographed using a gel documentation system. SYBR green dye was also used for the detection of amplicons using the naked eye. In the presence of positive DNA amplification SYBR green changes colour from orange to green. One microliter of SYBR green I dye was added to each tube containing LAMP products.

The specificity and sensitivity of the LAMP assay

To verify the specificity of the LAMP assay for the detection of *N. americanus* DNA, the LAMP primers specific for hookworm were tested using DNA from other related helminths i.e. *S. mansoni, A. lumbricoides* and *T. trichiura*. In order to determine the sensitivity of the LAMP assay, 10 ng/ul of *N. americanus* DNA was successively diluted 10 times by the addition of 1 ul of a 1/10 dilution of the previous concentration.

## Results

### Primer and temperature selection for the LAMP reaction

**Fig. 1:**
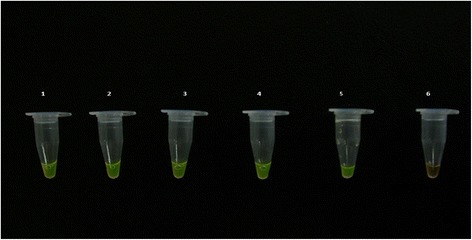
A photo showing the colour change using SYBR green nucleic acid stain as a method of visualizing LAMP amplicons. Samples 1 to 5 were positive for hookworms hence the observed colour change from orange to green, while sample 6 was the negative control thus there was no colour change

**Fig. 2:**
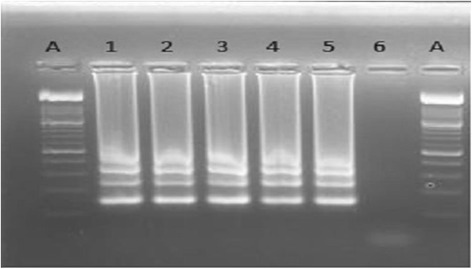
Agarose gel showing the fragments amplified from human faecal samples containing *N. americanus* DNA (lane 1–5). Lane 6 is the negative control (PCR water). A represents a 100-bp ladder

In this study, we designed 5 sets of primers for LAMP amplification. Among these primers, those that had the highest amplification rates and the shortest amplification time were selected for analysis (Table 1). To optimize the LAMP reaction temperature, we conducted the procedures under different temperature conditions. LAMP assays were performed under isothermal conditions between 59 °C and 66 °C. Based on the larger amounts of DNA amplicons and the optimal temperature for Bst DNA polymerase activity, we choose 63 °C as the final reaction temperature for our LAMP assay. Amplification was completed within 1 h isothermally at 63 °C in a water bath. The products of the LAMP reaction were detected by SBYR green nucleic acid stain where the positive samples changed colour from orange to green while the negative control remained orange (Fig. 1). The products were also viewed on a 2 % agarose gel and showed ladder-like patterns (Fig. 2).

### Specificity of the LAMP reaction

**Fig. 3:**
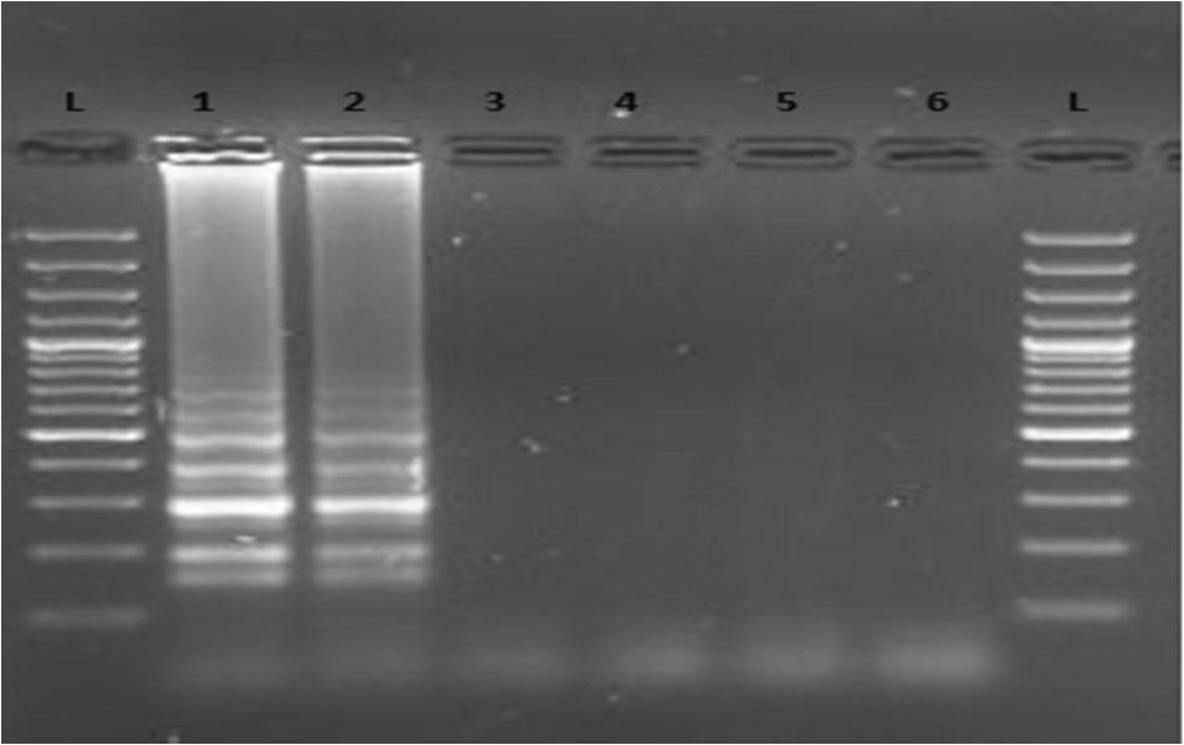
Agarose gel showing the specificity of the test, *N. americanus* DNA (lane 1 & 2), *S. mansoni* DNA (Lane 3), *A. lumbricoides DNA* (Lane 4), *T. trichiura* DNA (Lane 5) and Lane 6 is the negative control (PCR water). L represents a 100-bp ladder

To determine specificity of the primers, two hookworm isolates were subjected to the LAMP assay alongside samples positive by Kato-Katz technique for other helminths i.e. *S. mansoni, A. lumbricoides,* and *T. trichiura.* These were amplified using primers specific for hookworm (*N. americanus*). As shown (Fig. 3), LAMP products were amplified only from DNA samples of *N. americanus* and no false positive amplification was observed, indicating the high specificity of the established LAMP assays.

### Sensitivity of the LAMP reaction

**Fig. 4:**
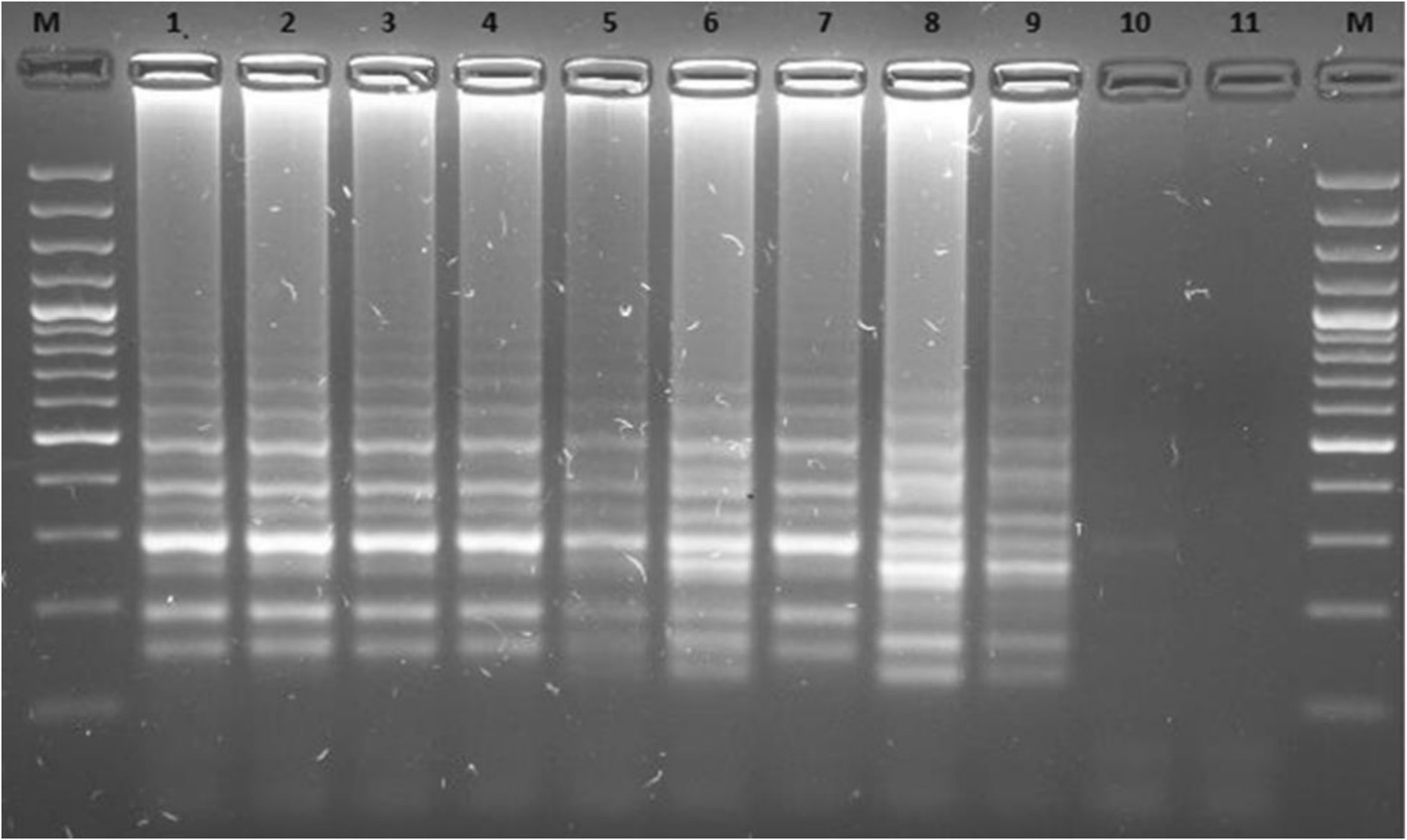
Agarose gel electrophoresis of loop-mediated isothermal amplification (LAMP) products from different concentrations of *Necator americanus* genomic DNA. Ten-fold serial dilutions starting with 10 ng of genomic DNA (lane 1) down to 0.4 fg (lane 10) were tested. Lane 11, negative control (no DNA); lane M molecular marker

Tenfold serial dilutions of total genomic DNA from *N. americanus* were used to test the sensitivity of the LAMP assay. The results indicated that the detection limit for the LAMP reaction was 0.4 fg of DNA (Fig. 4).

### Comparison of Loop mediated isothermal amplification (LAMP) against Kato-Katz

A total of 106 stool samples from the field were analysed and the results tabulated as shown (Table 2). Out of the 106 samples, 86 samples were found to be positive by Kato-Katz technique, while 83 of the positive samples were found to be positive by LAMP. 20 samples were found to be negative for hookworms by both Kato-Katz and LAMP techniques.

**Table 2:** Contingency table comparing LAMP technique with Kato-Katz (gold standard)

	+	–	Totals
+	83	0	83
–	3	20	23
Totals	86	20	106

LAMP (Screen test)

In terms of sensitivity LAMP showed 97 % while in terms of specificity LAMP showed 100 %. We also found the agreement between the two techniques by calculating the Kappa coefficient as described by Viera et al. [15], which is calculated as follows

$$ \mathrm{Kappa}\ \mathrm{coefficient} = \left(\%\ \mathrm{Observed}\ \mathrm{Agreement}.\right)\ \hbox{--}\ \left(\%\ \mathrm{agreement}\ \mathrm{Expected}\ \mathrm{b}\mathrm{y}\ \mathrm{chance}\right) = \mathbf{0.9} $$

$$ 100\ \% - \left(\%\ \mathrm{agreement}\ \mathrm{Expected}\ \mathrm{b}\mathrm{y}\ \mathrm{chance}\right) $$

The Kappa coefficient was 0.9, which shows an excellent agreement between the two techniques, thus proving LAMP to a reliable diagnostic tool for hookworms.

## Discussion

In this study, we reported on LAMP, a novel sensitive and rapid detection method for diagnosis of hookworm caused by *Necator americanus*. An accurate, specific and sensitive diagnosis system for hookworm is critical for accurate detection of hookworm following mass drug administration when infection intensities are lowered following chemotherapy. The current WHO recommended strategies in the control of schistosomiasis and STHs is by mass drug administration (MDA). One notable result of MDA is the lowering of infection intensity both in individuals and populations under treatment. This requires diagnostic methods that are sensitive enough to detect infections at very low intensities. Parasitological diagnostic methods such as Kato-Katz have commonly been used to diagnose STHs but these methods have the disadvantage of low sensitivity mainly due to non-random egg distribution in faecal samples, hence making the monitoring of the success of control programmes a challenge. Molecular based techniques that are dependent on Polymerase chain reaction (PCR) are available and are more sensitive and specific than the parasitological techniques. However, these methods are very expensive because of their requirement for costly equipment. Loop mediated isothermal amplification technique (LAMP) is a technique developed by Notomi et al., [7] that amplifies DNA with high specificity, efficiency and rapidity under isothermal conditions.

In this study we have developed a LAMP assay that can detect hookworm DNA in faecal human samples. The developed LAMP technique was based on the internal transcribed spacer 2 (ITS-2) of the nuclear ribosomal DNA. This region was chosen as a potential marker because of its low mutation rates within species and substantial differences between species [16], hence making it an excellent candidate for designing LAMP primers for detection of hookworms. The primers designed were highly specific and only amplified DNA of *N. americanus* and had no cross-reaction with nucleic acid from closely related species.

LAMP has the characteristics that make it ideal for field work and has already been used in rural laboratories in developing areas for the diagnosis of tuberculosis [17]. One advantage of a LAMP test over the conventional PCR is that sophisticated equipment required for PCR, is not required for the LAMP assay, a simple detection system based on staining the amplification products with SYBR green dye, which can be visualized with the naked eye, demonstrating the feasibility of using this assay under field conditions. In the present study, we were able to detect the amplified DNA not only using agarose gel but also with the naked eye following staining with SYBR green dye. Because the assay reaction is isothermal, a simple water bath can be used for this test. Furthermore, the fact that the developed assay could detect parasite DNA concentrations as low as 0.4 fg suggests that even low intensity of *N. americanus* infections can readily be detected using the developed LAMP assay. The Kato-Katz technique diagnosed three samples as positive but the same samples were negative by LAMP technique. We attributed this difference to human error since our control samples were always in agreement with the LAMP results. Kato-Katz technique relies on expert microscopy that is subjective and due to a number of factors such as technical experience, eye sight, preparation of the stool smears among other factors, therefore reading may differ from one reader to the other. Among the other tests available for the diagnosis of hookworms is the Mini-FLOTAC device which is the most promising. However, a recent meta-analysis and field studies comparing it’s sensitivity with that of Kato-Katz have shown no significant difference [18–20].

We recommend further development of the technique to make it adaptable for application in the field laboratory set up. Specifically, the DNA extraction kit should be replaced with a simpler and inexpensive DNA extraction method. Also the LAMP reagents would need to be premixed and ready to use to avoid too many pipetting steps that may result in sample contamination; moreover reagents that can be stored at room temperature need to be formulated as this could facilitate their viability under field-laboratory conditions.

## Conclusion

In summary, in the current study, we showed that the LAMP technique based on the ITS-2 region enables specific detection of *N. americanus* but excludes related helminth species. It is a novel technique that can potentially be used for rapid diagnosis of *N. americanus* infections, not only in laboratories but also in a field setting. In conclusion, the LAMP method described in this study represents a new, sensitive, specific, and rapid protocol for the detection of *N. americanus* and has the potential for point of care application. The findings of this present study demonstrate that this technique can be applied to epidemiological studies to detect hookworm prevalence and infection intensities in infected individuals.
